# Structure–Property Relationships and Thermal Degradation Mechanism of Terpene Methacrylate-Styrene Copolymers

**DOI:** 10.3390/ma19050974

**Published:** 2026-03-03

**Authors:** Marta Worzakowska

**Affiliations:** Department of Polymer Chemistry, Institute of Chemical Sciences, Faculty of Chemistry, Maria Curie Skłodowska University, Gliniana 33 Street, 20-614 Lublin, Poland; marta.worzakowska@mail.umcs.pl

**Keywords:** copolymers, citronellyl methacrylate, geranyl methacrylate, TG/FTIR, DSC

## Abstract

The ultraviolet (UV) copolymers of two monomers, one methacrylic and the other vinyl monomer (styrene, S) were prepared. As methacrylic monomers, citronellyl methacrylate (CM) or geranyl methacrylate (GM) were used. The preparation was proven to contain high solvent- and chemical-resistant copolymers due to their cross-linked structure with the conversion degree of the double bonds above 0.92 for poly(citronellyl methacrylate)/polystyrene (PCM/PS) and above 0.85 for poly(geranyl methacrylate)/polystyrene (PGM/PS) copolymers. The obtained copolymers showed only one glass transition temperature (*T*_g_). Depending on the structure and amount of the used methacrylic monomer, the *T*_g_ values were from 0.4 °C to −15.2 °C for PCM/PS copolymers and from −23.2 °C to −50.5 °C for PGM/PS copolymers. The thermogravimetric analysis (TG/DTG) showed a higher thermal stability for PCM/PS (148–187 °C) than for PGM/PS copolymers (119–159 °C) in inert and oxidative atmospheres. The simultaneous thermogravimetric analysis coupled with Fourier Transform Infrared spectroscopy (TG/FTIR) showed that the pyrolysis and oxidative decomposition of the tested copolymers took place according to the radical mechanism. This led to receiving a mixture of low molecular mass organic molecules containing saturated and unsaturated fragments, carbonyl groups, aromatic fragments as well as to CO, CO_2_ and H_2_O. This indicated the depolymerization process (inert) and further oxidation processes of the initially formed volatiles and/or residues in oxidative conditions.

## 1. Introduction

Styrene is a vinyl monomer, commonly, industrially applied for the production of a whole range of synthetic polymeric materials, including the formation of solid plastic and foam polystyrene. Among solid plastic forms, general purpose polystyrene and high-impact polystyrene are well known. The foam forms, expanded polystyrene and extruded polystyrene, are also mentioned [1,2,3,4,5,6]. In addition to creating homopolymers in various forms, styrene is also often used in compositions with other monomers to obtain copolymers in order to improve or modify polystyrene properties. Such a wide use of styrene is due to its high susceptibility to polymerization and thus obtaining materials with desirable beneficial properties for various practical applications. Such styrene materials are characterized by high rigidity, transparency, high flexural strength and surface hardness, high resistance to wear and tear, low density, and the ease with which they can be painted, printed, and colored. Styrene is a widely used in the production of plastics, synthetic rubbers, latex paints, coatings, polyesters, styrene-alkyd coatings, and other materials. Styrene-based polymeric materials are used as common plastics in packaging, insulation, and various consumer products; in automotive parts, appliance housings, and various industrial components; in fiber-reinforced polymer composites for applications like car parts, boat hulls, and bathroom fixtures; in tire manufacturing, shoe soles, and other rubber products; for pipes, roofing, and other building materials; for the production of toys, household appliances, and sporting goods, etc. [7,8,9,10,11,12,13,14,15,16,17,18,19]. However, polystyrene materials are also poorly recyclable and non-biodegradable. When exposed to heat, these materials can release harmful chemicals, posing risks in food contact or high-heat environments. Moreover, most of them are not resistant to contact with oils, solvents and strong acids or bases. Usually, commercial polystyrene materials show limited thermal resistance, poor ultraviolet (UV) and weather resistance, high moisture absorption or fragility. For these reasons, the properties of polystyrenes are often modified. One of the modification methods is to obtain styrene copolymers with another polymerizable monomer.

The most commonly known copolymers of styrene are copolymers with acrylonitrile, butadiene, isoprene, methyl methacrylate and acrylic monomers. A random copolymer of styrene-acrylonitrile (SAN) containing 70–80% mass styrene is characterized by improved strength, rigidity and chemical resistance compared to a styrene homopolymer. This copolymer is used, among others, in household goods, cosmetics packing and sanitary articles [20,21,22,23,24,25,26,27,28]. However, the copolymer of styrene and butadiene (SBR) with high styrene content (above 70% mass), due to its good abrasion and aging resistance, is used for the creation of shoe soles, automotive parts and various mechanical rubber goods. The other examples of copolymers are styrenic block copolymers including styrene-butadiene-styrene (SBS) and styrene-ethylene/butylene-styrene (SEBS) copolymers, which are characterized by rubber-like elasticity and thermoplastic processability. As a result, they are industrially used as adhesives, sealants and modified asphalts. Other styrene copolymers include materials such as styrene-co-butyl acrylate, potentially useful for fuel cell membranes, and styrene-isoprene-styrene, with thermoplastic elastomer properties. Terpolymers with high toughness and impact resistance, including styrene-acrylonitrile-butadiene or styrene-maleic anhydride copolymers used in various applications, were also described [29,30,31,32,33,34,35].

Despite the wide range of commercially available styrene-based materials used both in industry and everyday life, the new materials are still in demand. In particular, the materials with limited styrene content, due to its properties (volatilization and evaporation), and with the same or improved functional properties are desirable. The second aspect that is taken into account in the synthesis of new polymeric materials is the increase in their biodegradability. Usually, commonly used styrene-based materials are materials obtained from petrochemical reagents. Therefore, they are non-biodegradable materials which are most often collected in landfills. It takes hundreds of years for them to decompose there. For these reasons, intensive research is undertaken on polymeric materials based on natural compounds originating from renewable natural sources. Both monomers and polymers are used in these studies to obtain materials with properties comparable or better than those of known, petrochemical materials. Terpene compounds are one of the natural monomers that can be used in the copolymerization process with styrene. To date, the application of such terpenes, like *α*-pinene, *β*-pinene, limonene, *β*-myrcene, ocimene, *β*-farnesene, turpentiene, allocimene, linallol, nerol, geraniol, menthol, etc., is known. The known styrene-terpene copolymers form one of the classes of the bio-based materials that combine the rigidity of polystyrene with the elastomeric properties of terpene polymers. Such styrene-terpene copolymers are versatile, sustainable materials that, depending on the terpene compound used, are characterized by good adhesion, flexibility, resistance to atmospheric and chemical factors, low glass transition temperature, high transparency and good compatibility with various resins. As a result of their properties, they have a wide range of applications as binders in adhesives, components of paints, varnishes, asphalts and bituminous masses, etc. They are also used in the production of rubber, cosmetics and synthetic materials [36,37,38,39,40,41,42,43,44,45,46,47,48,49,50,51,52,53,54,55].

In the present work, it was proposed to use methacrylic monomers synthesized based on primary terpene alcohols such as citronellol and geraniol as a component of styrene copolymers. The copolymers were prepared in the ultraviolet (UV) polymerization process using different amounts of methacrylic monomers and styrene. The effect of the amount of a given monomer on the properties such as solubility in both polar and non-polar solvents, chemical resistance in acidic, alkaline and buffer environments, glass transition temperature determined according to the differential scanning calorimetry (DSC) method, thermal resistance evaluated based on the thermogravimetric/derivative thermogravimetric (TG/DTG) method and the decomposition course studied with the use of simultaneous thermogravimetric analysis coupled with the Fourier Transform Infrared spectroscopy (TG/FTIR) method of the obtained polymeric materials was discussed. The properties of the obtained copolymers were compared with the properties of homopolymers: polystyrene, poly(citronellyl methacrylate) and poly(geranyl methacrylate) obtained under the same experimental ultraviolet (UV) conditions.

## 2. Materials and Methods

### 2.1. Materials

Methacrylic monomers: Citronellyl methacrylate (CM) and geranyl methacrylate (GM) by acylation of terpene alcohol: citronellol (CAS no. 106.22-9, purity ≥ 95%) or geraniol (CAS no. 106-24-1, purity ≥ 97%) and methacryloyl chloride (CAS no. 920-46-7, purity 97%) in the presence of trimethylamine (CAS no. 121-44-8, purity ≥ 99.5%) were prepared according to Refs. [56,57,58]. The commercially available, aromatic vinyl monomer (styrene, S, CAS no. 100-42-5, purity ≥ 99%) was delivered by Sigma-Aldrich (St. Louis, MO, USA). The 2,2-dimethoxy-1,2-diphenylethan-1-one (initiator, CAS no. 24650-42-8, purity 99%) and the following solvents, methanol, butanol, acetone, acetic acid, ethyl acetate, tetrahydrofuran, diethyl ether, dioxane, chloroform, carbon tetrachloride, hexane, cyclohexane and toluene, were delivered by Merck (Darmstadt, Germany). Sodium hydroxide, hydrochloric acid and buffer solutions with pH 4, 7, and 10 were from Avantor Performance Materials (Poland S.A, Gliwice, Poland).

### 2.2. The Ultraviolet (UV) Polymerization

The appropriate compositions of the suitable methacrylic monomer, vinyl monomer and an initiator were exposed to UV irradiation, Table 1. UV irradiation was carried out using the TL20W/05 SLV low-pressure mercury lamp (Philips Lighting, Warsaw, Poland) with 340–365 nm. The power of the lamp was 20 W. The area of the irradiated surface was 0.0025 m^2^. The exposure conditions were as follows: irradiation time 10 min and irradiation temperature 25 °C. The obtained UV films were subjected to conditioning at 60 °C for 5 h and at 120 °C for 1 h.

### 2.3. Methods

#### 2.3.1. The Fourier Transform Infrared Spectroscopy (FTIR)

An FTIR Tensor 27 (Bruker, Brema, Germany) instrument equipped with a Digital TechTM detector system, high-sensitivity DLaTGS (Bruker, Brema, Germany), was used to collect the FTIR spectra. The FTIR spectra were collected from 600 cm^−1^ to 4000 cm^−1^ using KBr tablets. The resolution of each spectrum was 4 cm^−1^. The 62 scans per spectrum were gathered. The KBR tablets were prepared by mixing 3 mg of the tested material with 300 mg of KBr, grinding in an agate mortar and pressing on the Specac Atlas™ manual 15T hydraulic press (Specac Inc., Washington, PA, USA). The conversion degrees (α) of the C=C bonds were calculated based on the following equation:

α = 1 − (*R*_p_/*R*_m_) (1)

where *R*_m_ is the ratio of the C=C peak area integration to the C=O peak area integration for the monomer, and *R*_p_ is the ratio of the C=C peak area integration to the C=O peak area integration for the resulting polymer, respectively [58,59].

To calculate *R*_m_ and *R*_p_, the peak areas integration of the C=C stretching vibrations bands at 1636 cm^−1^ (methacrylic bonds) and at 1629 cm^−1^ (vinyl bonds) and at 1676 cm^−1^ (ethylenic bonds), and the peak area integration of the C=O stretching vibration band at 1718 cm^−1^ (citronellyl methacrylate) or 1724 cm^−1^ (geranyl methacrylate) visible in the FTIR spectra for the obtained monomers and polymers, were used, respectively.

#### 2.3.2. The Solubility

The gravimetric method was used at 25 °C to evaluate the solubility of the tested materials. A total of 0.2 g of a sample was immersed in the appropriate solvent. The solubility tests were conducted over a period of six months. At the specific time intervals, the solvent was poured off, and the sample was dried and weighed. Both polar and non-polar solvents were used. As solvents, water, methanol, butanol, acetone, acetic acid, ethyl acetate, tetrahydrofuran, diethyl ether, dioxane, chloroform, carbon tetrachloride, hexane, cyclohexane and toluene were used. 

#### 2.3.3. The Chemical Stability

The gravimetric method was used at 25 °C to examine the chemical stability of the prepared materials. A total of 0.2 g of a sample was immersed in the chosen solutions (1 M NaOH, buffer solutions with pH 4, 7, and 10, and 1 M HCl). The chemical stability tests were carried out over a period of six months. At specific time intervals, the samples were washed with distilled water, dried and weighed. The solubility and chemical stability of the tested polymers as a percentage change in mass (Δ*w*_S_) was calculated from the following [60]:

**Δ*w*_S_** = (*m*_1_−*m*_2_)/*m*_1_ × 100% (2)

*m*_1_—the sample mass before the solubility or chemical stability test, *m*_2_—the sample mass after the solubility or chemical stability test, and Δ*w*_S_—the percentage change in mass.

#### 2.3.4. The Differential Scanning Calorimetry (DSC)

The DSC method (a DSC 204 Jupiter instrument, Netzsch, Germany) was used to evaluate the glass transition temperature (*T*_g_) of the prepared polymeric materials. The 10 mg of sample was placed in an Al crucible with a pierced lid. Each sample was heated from −120 °C to 150 °C, and then cooled to −120 °C and reheated to 150 °C in argon as a furnace atmosphere. The heating rate was 10 °C/min. The flow rate of argon was 40 mL/min. The values of the glass transition temperature (*T*_g_) from the second DSC scan were evaluated.

#### 2.3.5. The Thermogravimetric/Derivative Thermogravimetric/Differential Scanning Calorimetry Analysis (TG/DTG/DSC)

The TG/DTG/DSC method was used in order to study the thermal properties of the tested polymers. An STA 449 Jupiter F1 instrument produced by Netzsch, Selb, Germany, was used. The 10 mg of sample was placed in an open Al_2_O_3_ crucible. The sample was heated from 40 °C to 550 °C with a heating rate of 10 °C/min. As a furnace atmosphere, helium with a flow rate of 40 mL/min and synthetic air with a flow rate of 100 mL/min were applied. The thermal stability of the materials was described as the temperature at which there is a mass loss of 5% (*T*_5%_), and maximum decomposition temperatures (*T*_max_), mass loss in each decomposition stage (Δ*m*) and residual masses (*m*_r_) were determined.

#### 2.3.6. The Simultaneous Thermogravimetric Analysis Coupled with the Fourier Transform Infrared Spectroscopy (TG-FTIR)

The volatiles released during the decomposition of the prepared polymeric materials by the simultaneous TG-FTIR analysis were defined. An STA 449 Jupiter F1 instrument produced by Netzsch, Selb, Germany, connected online to a Fourier Transform Infrared Spectroscopy (FTIR) analyzer (FTIR TGA 585, Bruker, Brema, Germany), was applied. The FTIR analyzer, through a Teflon tube (a diameter 2 mm) heated to 200 °C, was connected online to an STA device. The FTIR spectra of the volatiles in the range of 600–4000 cm^−1^ were collected. The resolution of each spectrum was 4 cm^−1^. The gaseous FTIR spectra for the samples heated in inert (a helium atmosphere, a flow rate of 40 mL/min) and in oxidizing (synthetic air, a flow rate of 100 mL/min) conditions were gathered.

## 3. Results and Discussion

### 3.1. The Fourier Transform Infrared Spectroscopy (FTIR) Results

**Figure 1:**
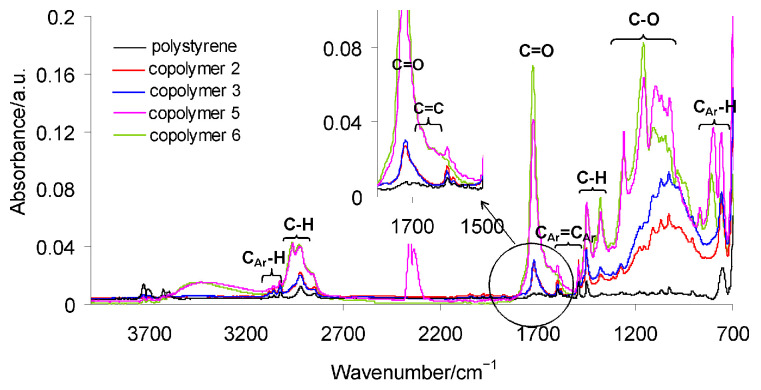
The Fourier Transform Infrared (FTIR) spectra for the tested materials.

**Scheme 1:**
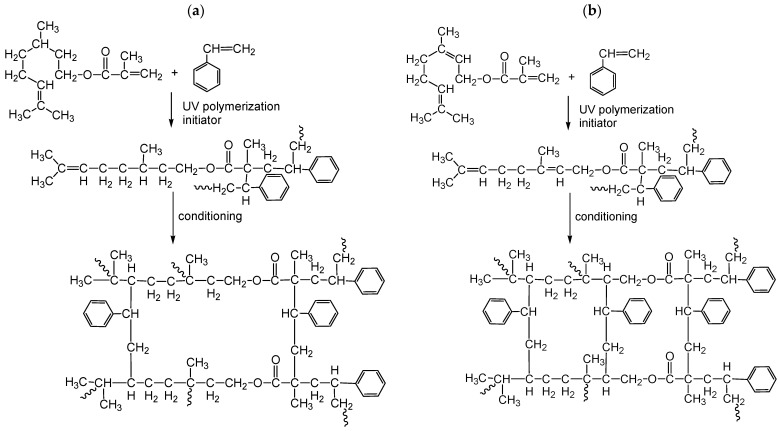
The theoretical structure of the prepared polymeric materials: (**a**) citronellyl methacrylate/styrene copolymers and (**b**) geranyl methacrylate/styrene copolymers.

The FTIR spectra for the selected polymer materials are shown in Figure 1. As is clearly visible, all of the absorption bands connected with the characteristic group vibrations present in the chemical structure of the prepared polymeric materials appear. The occurrence of the absorption bands at 3024–3093 cm^−1^ (the C_Ar_-H stretching vibrations), 2840–2918 cm^−1^ (the C-H stretching vibrations), 1718 cm^−1^ (the C=O stretching vibrations in citronellyl methacrylate-based polymeric materials), 1724 cm^−1^ (the C=O stretching vibrations in geranyl methacrylate-based polymeric materials), 1490–1600 cm^−1^ (the C_Ar_=C_Ar_ stretching vibrations), 1377–1450 cm^−1^ (the C-H deformation vibrations), 1010–1200 cm^−1^ (the C-O stretching vibrations) and 756–960 cm^−1^ (the C_Ar_-H out-of-plane deformation vibrations) clearly confirms their structures [56,57,58,61]. What is noteworthy is that the C=C stretching vibrations at 1629 cm^−1^ due to the vibrations of vinyl bonds of styrene are not observed in the FTIR spectra for copolymers. Moreover, in the case of polymeric materials prepared with the use of citronellyl methacrylate, there is no absorption band at 1636 cm^−1^, characteristic for a methacrylic bond. Also, the absorption band at 1676 cm^−1^ (due to ethylenic bonds from citronellyl methacrylate), as a residual band with very little intensity, is observed [61]. For the polymeric materials obtained with the use of geranyl methacrylate, low-intensity absorption signals responsible for the C=C stretching vibrations at 1636 cm^−1^ (methacrylic bond) and at 1676 cm^−1^ (ethylenic bonds) are still present in the FTIR spectra. Hence, it can be suspected that geranyl methacrylate is a monomer with lower reactivity in the UV polymerization process with styrene compared to the reactivity of citronellyl methacrylate. However, it is clearly seen that all types of the C=C double bonds take part in the UV polymerization process, but their conversion is different and depends on the methacrylic monomer used. Table 2 contains the calculated conversion degrees of the C=C double bonds based on the FTIR spectra. The conversion degree of the C=C double bonds (α) for geranyl methacrylate/styrene copolymers is lower as compared to the α for citronellyl methacrylate/styrene copolymers. This is due to the higher reactivity of the citronellyl methacrylate monomer containing only one ethylenic bond in its structure. Thus, the formation of a less cross-linked structure than for the geranyl methacrylate monomer containing two ethylenic bonds is expected. Lower cross-linked density is associated with the greater flexibility of the prepared polymer networks. As is expected and confirmed from the FTIR spectra, two ethylenic bonds of the geranyl methacrylate monomer take part in the UV polymerization process. Thus “more dense” gels are formed at the beginning of the polymerization process. This makes it difficult to react all of the available double bonds. Therefore, some of them remain unchanged in the final cross-linked product. Scheme 1 shows the theoretical structure of the prepared materials.

### 3.2. The Solubility of the Tested Polymers Results

The solubility results of the tested polymers in the solvents of different polarities and chemical structures are presented in Table 3 and Table 4. Due to the fact that the obtained methacrylic-based copolymers are cross-linked materials, it is expected that they will be characterized by reduced solubility. The obtained results confirm this assumption. The solubility of both homopolymers (PCM and PGM) and PCM/PS and PGM/PS copolymers is very low; the determined mass loss is below 1%. Moreover, the obtained materials do not swell in the used solvents. As is clearly visible, the polystyrene homopolymer is a soluble material in some of the solvents used. The very low solubility of the obtained copolymers additionally confirms the absence of styrene homopolymer in their composition. This also proves that the applied UV polymerization process conditions allow for the effective incorporation of the styrene into the copolymer’s structure.

### 3.3. The Chemical Stability Results

The chemical stability results are shown in Table 5. In this case, there is also an almost complete lack of influence of a specific chemical environment on the change in the mass of the tested materials. The percentage change in mass is below 0.5%. This indicates that the chemical environments used, alkaline, neutral and acidic, do not cause chemical degradation of the obtained materials, or their swelling. This confirms that the tested polymeric materials have excellent chemical stability. The chemical environment used does not cause degradation or hydrolysis, due to their high degree of cross-linking.

### 3.4. The Differential Scanning Calorimetry (DSC) Results

**Figure 2:**
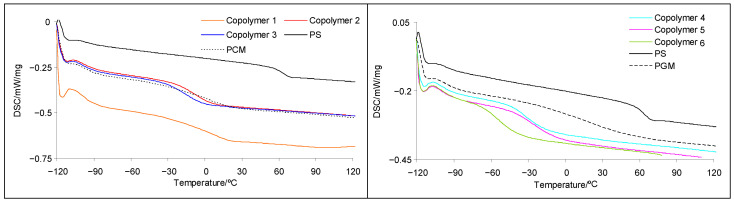
The glass transition temperature (*T*_g_) evaluated by DSC.

A DSC method was used to evaluate the glass transition temperatures (*T*_g_) of the tested polymeric materials. The DSC curves are shown in Figure 2. In addition, Table 6 presents the *T*_g_ values. The *T*_g_ values for citronellyl methacrylate/styrene copolymers are from 0.4 °C to −15.2 °C. However, the *T*_g_ values for geranyl methacrylate/styrene copolymers are lower and range from −23.2 °C to −50.5 °C. Taking into account the conversion degree of the double bonds determined based on the FTIR, it can be stated that higher *T*_g_ values for copolymers obtained using citronellyl methacrylate are caused by a higher number of cross-links in the obtained polymer networks as compared to the geranyl methacrylate-based copolymers.

In addition, it is noticed that with the increase in methacrylic monomer content in the copolymers, the *T*_g_ values decrease. This is most likely caused by the extension of aliphatic chains macromolecules, which gives them greater flexibility compared to copolymers containing larger amounts of styrene as an aromatic monomer. Moreover, it can be said that the use of compositions containing citronellyl or geranyl methacrylate monomers and styrene results in the preparation of polymeric materials with more elastomeric and flexible properties.

### 3.5. The Thermogravimetric/Derivative Thermogravimetric/Differential Scanning Calorimetry (TG/DTG/DSC) Results (Inert Conditions)

**Figure 3:**
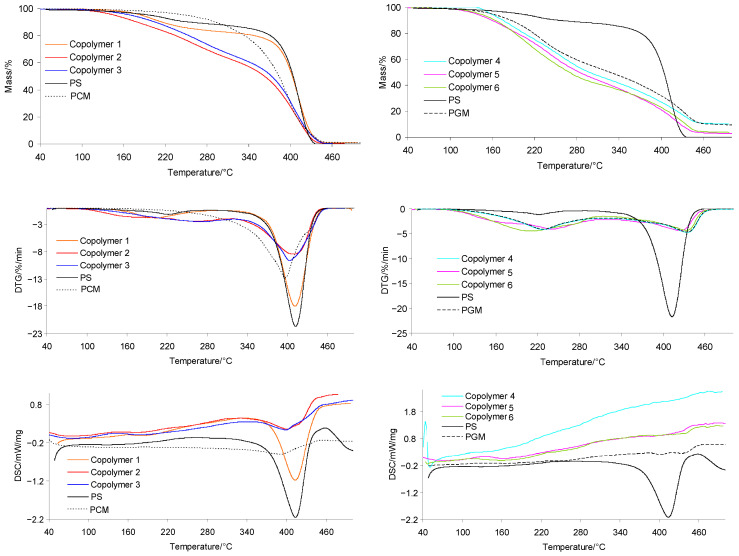
The thermogravimetric/derivative thermogravimetric/differential scanning calorimetry (TG/DTG/DSC) curves (helium).

Figure 3 shows the TG/DTG/DSC curves. In addition, Table 7 presents the collected data read from the TG/DTG curves. A slightly higher thermal stability is observed for the citronellyl methacrylate/styrene copolymers compared to geranyl methacrylate/styrene copolymers. The thermal stability marked as 5% of mass loss is from 148 °C to 187 °C for citronellyl methacrylate-based copolymers and from 142 °C to 159 °C for geranyl methacrylate-based copolymers. All of the tested copolymers decompose in at least two stages in helium atmosphere. The first decomposition stage spreads from *T*_5%_ to 310–320 °C. This stage is depicted by a “fuzzy” DTG signal with one maximum at 205–277 °C (*T*_max1_) for citronellyl methacrylate-based materials or two maxima, one at 180–212 °C (*T*_max1_) and the other at 238–276 °C (*T*_max1a_), for geranyl methacrylate-based copolymers. The mass loss (Δ*m*_1_) is from 17.4% to 38% for copolymers 1–3 (citronellyl methacrylate-based copolymers) and from 54.1% to 59.4% for copolymers 4–6 (geranyl methacrylate-based copolymers) during the first stage of decomposition. The second decomposition stage spreads from ca. 310–320 °C to 460 °C (copolymers 1–3) or to 500 °C (copolymers 4–6). In addition, the thermal resistance of the tested copolymers is lower than homopolymers (PGM, PCM and PS). This may indicate their presence at the ends of a cross-linked network of linear/aliphatic groups containing unreacted double bonds in their structure. These groups promote the thermal depolymerization of the tested copolymers and thus lower the initial decomposition temperature. Such a structure can be compared to a ball with threads attached to it, where the ball is a cross-linked network and the threads are linear aliphatic chains containing saturated and unsaturated fragments. In addition, it is clearly visible that the copolymers containing 50% mass of methacrylic monomer and 50% mass of styrene show the lowest thermal stability of all obtained copolymers.

### 3.6. The Simultaneous Thermogravimetric Analysis Coupled with the Fourier Transform Infrared Spectroscopy (TG/FTIR) Results (Inert Conditions)

**Figure 4:**
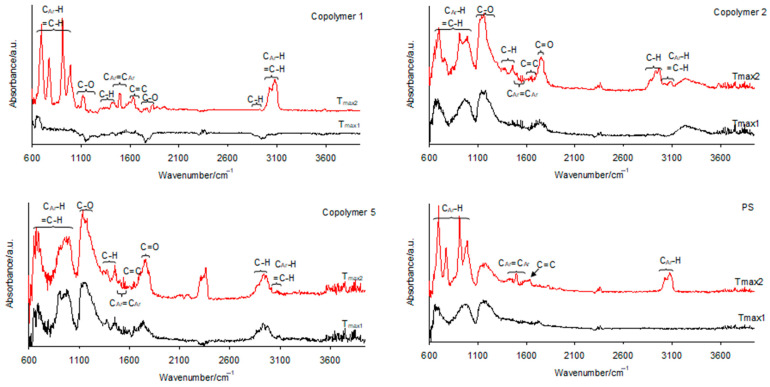
The gaseous Fourier Transform Infrared (FTIR) spectra collected at *T*_max1_ and *T*_max2_ (helium).

Figure 4 shows the gaseous FTIR spectra gathered at the characteristic maximum decomposition temperatures (*T*_max1_, *T*_max1a_ and *T*_max2_) for the selected PCM/PS and PGM/PS copolymers. In addition, the FTIR spectra of the emitted volatiles from PS are provided for comparision. As is already known, the pyrolysis of polystyrene includes radical reactions, e.g., random-scission and end-chain *β*-scission processes in which mainly the depolymerization product—styrene—is formed. However, other radical processes may occur for the generation of volatiles such as benzene, toluene and ethylbenzene [61,62,63,64,65]. The obtained experimental gaseous FTIR spectra collected during the heating of PS indicate very low emission of volatiles at *T*_max1_ due to low mass loss (11.6%) at this stage. However, the presence of the C-H out-of-plane bending of the aromatic ring (695–991 cm^−1^) and aromatic ring vibrations (1563–1700 cm^−1^) can be seen in this spectrum. This indicates a small emission of aromatic compounds because of the decomposition of macromolecules with a lower double bond conversion and thus lower molecular masses. If the furnace temperature exceeds 280 °C, a significant increase in volatile emission connected with the main decomposition stage of PS is observed. The recorded gaseous FTIR spectrum at this stage of PS decomposition clearly indicates the emission of its depolymerization products [66,67,68,69,70].

If we analyze the decomposition course of the tested copolymers, we can notice that in the case of PCM/PS copolymers, the greatest intensity of the released gaseous decomposition products is observed at *T*_max2_ (copolymer 1 and copolymer 2, Figure 4). Taking into account the composition of the copolymers, the highest intensity of absorption signals is observed with the monomer used in larger amounts for the polymerization process. For copolymer 1, containing 80% mass styrene and 20% mass citronellyl methacrylate, the dominant absorption bands originating from the decomposition products of polystyrene mers incorporated into the copolymer structure are observed. However, the decomposition of copolymer 2 (50% mass styrene: 50% mass citronellyl methacrylate), results in the appearance of the overlapping or slightly different wavenumber values for absorption bands of the FTIR spectra collected at *T*_max2_ due to the emission of gaseous decomposition products of both polystyrene and poly(citronellyl methacrylate) mer units. This confirms that the bonds in the formed polymer network are characterized by similar decomposition energy. The determined mass loss during the second decomposition stage (Δ*m*_2_) proves that this stage is the main decomposition stage of PCM/PS materials. The appearance of the following absorption bands at 692–989 cm^−1^ (the =C-H and the =C_Ar_-H out-of-plane deformation vibrations), 1039–1224 cm^−1^ (the C-O stetching vibrations), 1322–1445 cm^−1^ (the C-H deformation vibration, respectively), 1500–1581 cm^−1^ (the C_Ar_=C_Ar_ the stretching vibrations), 1626–1654 cm^−1^ (the C=C stretching vibrations), 1733–1825 cm^−1^ (the C=O stretching vibrations), 2925–2965 cm^−1^ (the C-H stretching vibrations) and 3022–3066 cm^−1^ (the C=C and C_Ar_-H stretching vibrations) clearly indicates the creation of mainly depolymerization products of copolymer 1. Among the emitted volatiles, mainly styrene and low amounts of citronellyl methacrylate are expected. The emission of styrene is predominant since this copolymer is formed from 80% mass of styrene and only 20% mass of citronellyl methacrylate. Meanwhile, in the case of copolymer 2, the appearance of the FTIR spetrum is different. In this spectrum, the emission of the decomposition products of citronellyl methacrylate mer units increases compared to the products of decomposition of styrene mer units. This is due to the higher content of citronellyl methacrylate used to prepare this copolymer. The FTIR spectrum collected for copolymer 2 at *T*_max2_ clearly shows the presence of the following absorption peaks: 651–993 cm^−1^ (the =C-H and =C_Ar_-H out-of-plane deformation vibrations), 1124–1210 cm^−1^ (the C-O stetching vibrations), 1350–1470 cm^−1^ (the C-H deformation vibration, respectively), 1540–1600 cm^−1^ (the C_Ar_=C_Ar_ the stretching vibrations), 1640–1667 cm^−1^ (the C=C stretching vibrations), 1730–1810 cm^−1^ (the C=O stretching vibrations), 2865–2980 cm^−1^ (the C-H stretching vibrations), and 3054–3090 cm^−1^ (the C=C and C_Ar_-H stretching vibrations) [61]. This proves volatile emission from both styrene and citronellyl methacrylate units. It additionally confirms that all bonds present in the copolymer structure are characterized by similar decomposition energy.

Similar observations can be made in the case of PGM/PS copolymers. Higher emissions of the gaseous decomposition products are visible in *T*_max2_ compared to *T*_max1_. Both recorded FTIR spectra show the absorption peaks in the same wavenumbers. This confirms that the same chemical bonds from the structure of copolymers are decomposed at both maximum temperatures. The presence of the following absorption peaks in the FTIR spectra is clearly visible: 720–981 cm^−1^ (the =C-H and the =C_Ar_-H out-of-plane deformation vibrations), 1083–1284 cm^−1^ (the C-O stretching vibrations), 1363–1445 cm^−1^ (the C-H deformation vibration, respectively), 1506–1600 cm^−1^ (the C_Ar_=C_Ar_ the stretching vibrations), 1640–1680 cm^−1^ (the C=C stretching vibrations), 1720–1805 cm^−1^ (the C=O stretching vibrations), 2910–2980 cm^−1^ (the C-H stretching vibrations), and 3035–3070 cm^−1^ (the C=C and C_Ar_-H stretching vibrations). In addition, in the gaseous FTIR spectra collected at *T*_max2_, the absorption peaks characteristic for CO (the absorption bands at 2086 cm^−1^ and 2160 cm^−1^) and CO_2_ (the absorption bands at 2306–2354 cm^−1^) can also be seen, as indicated in Figure 4 [61,71,72,73].

In summary, it is stated that at lower temperatures (*T*_max1_), the C-C and C-O bonds present at the ends of polymer macromolecules (as branches with an ester moiety) undergo pyrolysis and/or macromolecules with lower molar mass undergo depolymerization. As the heating temperature increases, the breaking of the C-C and C-O bonds in the main macromolecular chains and in the branched chains connected with the reactions between the formed fragments are the most expected. Among the emitted volatiles, saturated, unsaturated, carbonyl and aromatic-type compounds are observed. This indicates that the decomposition of the tested copolymers occurs according to the radical mechanism. This process proceeds in an uncontrolled manner and thus a mixture of volatiles is formed. It should be remembered that the decomposition reactions taking place according to the radical mechanism lead to the production of the highly reactive radicals that can react with other radicals in an uncontrolled and random manner. This results in the formation of intermediate products, radicals with larger molecular masses and thus, various decomposition products.

### 3.7. The Thermogravimetric/Derivative Thermogravimetric/Differential Scanning Calorimetry (TG/DTG/DSC) Results (Oxidative Conditions)

**Figure 5:**
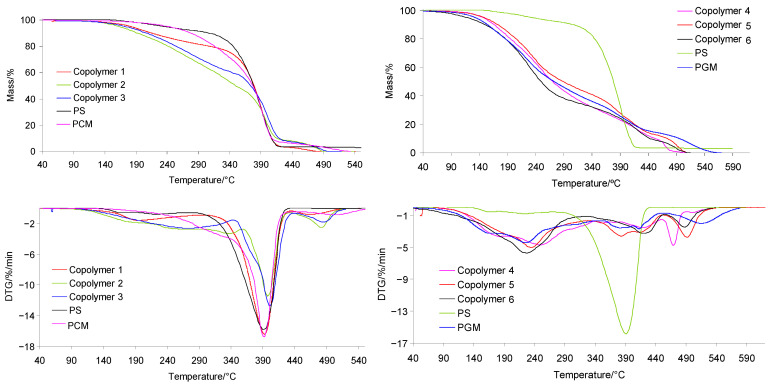

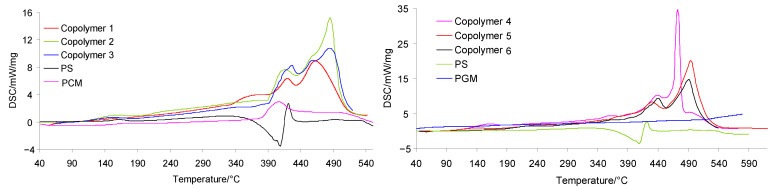
The thermogravimetric/derivative thermogravimetric/differential scanning calorimetry (TG/DTG/DSC) curves (synthetic air).

Figure 5 presents the TG/DTG/DSC curves collected in oxidative conditions. In addition, Table 8 shows the data determined from the TG/DTG curves. Similar to what is visible in an inert condition, the copolymers based on citronellyl methacrylate and styrene show slightly better thermal resistance as compared to the copolymers formed from geranyl methacrylate and styrene. The thermal stability is from 163 °C to 181 °C for citronellyl methacrylate-based coolymers and from 119 °C to 149 °C for geranyl methacrylate-based copolymers. All of the tested copolymers decompose in at least three main stages under the influence of the oxidizing conditions (oxygen from synthetic air furnace atmopshere). The first decomposition stage includes at least two steps (*T*_max1_, *T*_max1a_). The first stage of decomposition spreads from *T*_5%_ to 320–340 °C and similarly to a non-oxidizing atmosphere; it is described by a “fuzzy” DTG signal with two main maxima at *T*_max1_ and *T*_max1a_ between 182 and 249 °C for the tested copolymers. The mass loss (Δ*m*_1_) is from 17.9% to 53% for copolymers 1–3 (citronellyl methacrylate-based copolymers) and from 57% to 75.4% for copolymers 4–6 (geranyl methacrylate-based copolymers). The second stage of decomposition appears from ca. 320–340 °C to 440–450 °C and it is connected with the mass loss (Δ*m*_2_) from 39.1% to 78.1% for copolymers 1–3 and from 14.2% to 29.4% for copolymers 4–6. Moreover, in an oxidative atmosphere, an additional third decomposition stage above 440–450 °C with *T*_max3_ at 464–501 °C and a mass loss (Δ*m*_3_) between 4% and 13.6% is observed.

All of the tested copolymers decompose fully in oxidative conditions. If one looks closely at the course of the DSC curves, one can determine the presence of mainly exothermic signals. These exothermic DSC signals directly point to chemical reactions occurring under the heating and decomposition of the copolymers in the presence of oxygen. Among chemical reactions, oxidation, combustion and reactions of the formed radicals with oxygen can be mentioned. These may lead to the formation of different gaseous products during oxidative decomposition as compared to the gases released during heating of the prepared materials in an atmosphere without access to oxygen.

### 3.8. The Simultaneous Thermogravimetric Analysis Coupled with the Fourier Transform Infrared Spectroscopy (TG/FTIR) Results (Oxidative Conditions)

**Figure 6:**
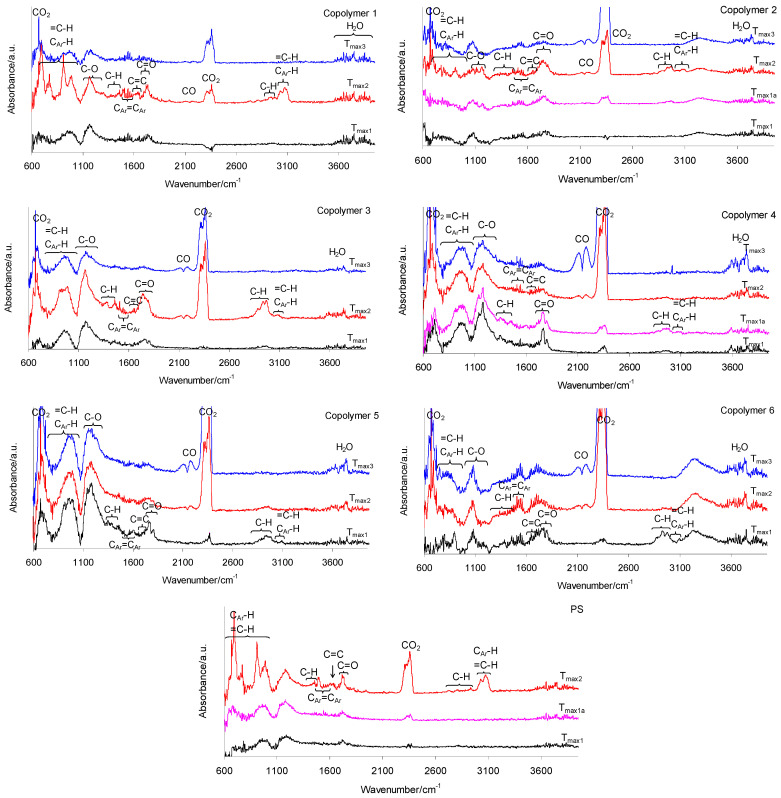
The gaseous Fourier Transform Infrared (FTIR) spectra collected at *T*_max1_, *T*_max2_ and *T*_max3_ (synthetic air).

Figure 6 presents the FTIR spectra for the emitted volatiles in a synthetic air atmosphere. As is clearly visible, the emission of the volatiles for copolymers 1–3 starts from *T*_5%_. The maximum emission at *T*_max2_ is observed. The FTIR spectra collected at *T*_max1_ and *T*_max2_ for PCM/PS copolymers (copolymers 1–3) show the appearance of absorption bands at the following: 678–975 cm^−1^ (the =C-H and the =C_Ar_-H out-of-plane deformation vibrations), 1060–1284 cm^−1^ (the C-O stetching vibrations), 1368–1476 cm^−1^ (the C-H deformation vibration, respectively), 1490–1556 cm^−1^ (at the low-frequency end, mixed C-H deformation vibrations and the C_Ar_=C_Ar_ stretching vibrations; at the high-frequency end, the C_Ar_=C_Ar_ the stretching vibrations), 1635–1670 cm^−1^ (the C=C stretching vibrations), 1700–1752 cm^−1^ (the C=O stretching vibrations), 2709 and 2788 cm^−1^, 2860–2954 cm^−1^ (the C-H stretching vibrations) and 3021–3073 cm^−1^ (the C=C and C_Ar_-H stretching vibrations). Also, the absorption bands responsible for CO_2_, CO and H_2_O emission at *T*_max1_ and *T*_max2_ are present. In turn, at *T*_max3_, mainly the formation of CO_2_ (the absorption bands at 2306–2354 cm^−1^), CO (the absorption bands at 2086 cm^−1^ and 2160 cm^−1^) and H_2_O (the absorption bands at 1400–1700 cm^−1^ and above 3500 cm^−1^) is confirmed. The PGM/PS copolymers (copolymers 4–6) at *T*_max1_ show the maximum intensity of the volatile emission. This is consistent with the amount of mass loss at this decomposition stage. A detailed analysis of the FTIR spectra at this decomposition stage confirms the presence of the absorption peaks at 694–995 cm^−1^ (the =C-H and =C_Ar_-H out-of-plane deformation vibrations), 1167–1284 cm^−1^ (the C-O stretching vibrations), 1457–1488 cm^−1^ (the C-H deformation vibration, respectively), 1517–1600 cm^−1^ (the C_Ar_=C_Ar_ the stretching vibrations), 1623–1650 cm^−1^ (the C=C stretching vibrations), 1681–1800 cm^−1^ (the C=O stretching vibrations), 2720–2780 cm^−1^ (the C-H stretching vibrations in aldehyde groups), 2933–2985 cm^−1^ (the C-H stretching vibrations), 3025–3068 cm^−1^ (the C=C and C_Ar_-H stretching vibrations) and the characteristic peaks for CO_2_, CO and water vapour formation. This proves volatile emission from both styrene and citronellyl methacrylate units. It additionally confirms that all bonds present in the copolymer structure are characterized by similar decomposition energy. At *T*_max2_, the intensity of the above-mentioned absorption bands is definitely decreased. In addition, at *T*_max3_, the emisssion of CO_2_, CO and H_2_O is dominant and clearly visible [61,71,72,73].

If one compares the FTIR spectra collected in an oxidizing atmosphere with the FTIR spectra collected in an inert atmosphere for both types of copolymers, one can see some differences in the wavenumber values for the organic gaseous decomposition products. These differences in the structure of the emitted gases are connected with the existence of oxygen in the atmosphere of the flowing gas through the furnace. As a result, formed organic gaseous decompostion products can further undergo chemical reactions such as oxidation or combustion (radical type reactions) under heating. This results in a mixture of gaseous products with a more diverse structure compared to those observed in inert atmosphere.

## 4. Conclusions

Ultraviolet (UV) copolymers based on different contents of citronellyl methacrylate, geranyl methacrylate and styrene were prepared. The Fourier Transform Infrared (FTIR) spectra confirmed their structure and the high conversions of the double bonds in the UV polymerization process. All of the obtained copolymers showed high solvent resistance and high chemical stability in environments with different pH. The cross-linked materials with glass transition temperatures from 0.4 °C to −50.5 °C, depending on the methacrylate monomer structure used and the content of UV composition, were prepared. The monomer type used and the applied thermogravimetric (TG) furnace atmosphere directly influenced the thermal stability of these copolymers. Generally, the prepared UV copolymers were more thermally stable in an inert atmosphere than in an oxidative atmosphere. Moreover, the thermal stability of UV copolymers based on citronellyl methacrylate was comparable or slightly higher than for geranyl methacrylate copolymers. This was due to the higher double bonds conversion and thus higher cross-linking density of the citronellyl methacrylate/styrene copolymers. The collected FTIR spectra of volatiles emitted under the heating of the copolymers confirmed the creation of a mixture of mainly organic volatiles. This was related to the bond cleavage from the structure of the prepared polymeric materials according to a radical mechanism. The studies performed showed that the use of methacrylic monomers prepared from terpene alcohols (citronellol and geraniol) in the mixture with styrene allowed for the production of cross-linked polymeric materials with high resistance to solvents and chemical environments and with glass transition temperatures below room temperature and with a satisfactory thermal resistance. Due to their properties and incorporation of monomers based on naturally occurring terpene alcohols, such materials can be suitable for applications where objects made of such copolymers are in direct and long-term contact with bases, acids, buffers and solvents of various polarities.

## Figures and Tables

**Table 1:** Composition of copolymers prepared with ultraviolet (UV) irradiation.

Polymer Name	Citronellyl Methacrylate (CM) [g]	Geranyl Methacrylate (GM) [g]	Styrene (S) [g]	Mass Ratio of Monomers (CM or GM to ST) [%]
PCM	3.0	-	-	100:0
PS	-	-	3.0	0:100
PGM	-	3.0	-	100:0
Copolymer 1	0.6	-	2.4	20:80
Copolymer 2	1.5	-	1.5	50:50
Copolymer 3	2.4	-	0.6	80:20
Copolymer 4	-	0.6	2.4	20:80
Copolymer 5	-	1.5	1.5	50:50
Copolymer 6	-	2.4	0.6	80:20

PCM—poly(citronellyl methacrylate), PGM—poly(geranyl methacrylate), and PS—polystyrene.

**Table 2:** The double bonds conversion degree (α).

α								
PCM	PS	PGM	Copolymer 1	Copolymer 2	Copolymer 3	Copolymer 4	Copolymer 5	Copolymer 6
0.90	0.93	0.88	0.92	0.94	0.93	0.85	0.85	0.86

**Table 3:** Percentage change in mass (Δ*w*_S_).

Polymer Name	Δ*w*_S_ [%]						
	Water	Methanol	Butanol	Acetone	Acetic Acid	Ethyl Acetate	Tetrahydrofuran
PCM	0.4	0.3	0.3	0.5	0.9	0.7	0.6
PS	0	0	0	s	0	s	s
PGM	0.6	0.7	0.9	0.4	0.8	0.8	0.7
Copolymer 1	0.8	0.8	0.7	0.5	0.6	0.7	0.3
Copolymer 2	0.7	0.8	0.7	0.5	0.5	0.7	0.4
Copolymer 3	0.7	0.7	0.8	0.6	0.4	0.7	0.5
Copolymer 4	0.9	0.9	0.8	0.6	0.5	0.6	0.4
Copolymer 5	0.8	0.9	0.7	0.5	0.5	0.7	0.7
Copolymer 6	0.7	0.8	0.7	0.4	0.4	0.6	0.5

where: s—soluble.

**Table 4:** Percentage change in mass (Δ*w*_S_).

Polymer Name	Δ*w*_S_ [%]						
	Diethyl Ether	Dioxane	Chloroform	CCl_4_	Hexane	Cyclohexane	Toluene
PCM	0.3	0.9	0.6	0.6	0.8	0.6	0.7
PS	0	s	s	s	swell	s	s
PGM	0.8	0.8	0.9	0.8	0.9	0.7	1.0
Copolymer 1	0.6	0.6	0.5	0.5	0.5	0.7	0.8
Copolymer 2	0.6	0.5	0.5	0.4	0.6	0.6	0.9
Copolymer 3	0.6	0.6	0.6	0.6	0.5	0.5	0.7
Copolymer 4	0.8	0.7	0.7	0.5	0.5	0.6	0.8
Copolymer 5	0.8	0.6	0.7	0.6	0.4	0.5	0.8
Copolymer 6	0.6	0.7	0.5	0.6	0.5	0.5	0.7

where: s—soluble.

**Table 5:** Percentage change in mass (Δ*w*_S_).

Polymer Name	Δ*w*_S_ [%]				
	1 M NaOH	1 M HCl	Buffer (pH 4)	Buffer (pH 7)	Buffer (pH 10)
PCM	0.4	0.0	0.0	0.1	0.1
PS	0	−2.0	−1.0	0	0
PGM	0.5	0.5	0.4	0.4	0.5
Copolymer 1	0.3	0.0	0.0	0.2	0.1
Copolymer 2	0.2	0.1	0.1	0.3	0.2
Copolymer 3	0.3	0.2	0.0	0.4	0.4
Copolymer 4	0.4	0.4	0.4	0.5	0.5
Copolymer 5	0.4	0.3	0.4	0.4	0.5
Copolymer 6	0.3	0.4	0.5	0.5	0.3

**Table 6:** The glass transition temperature (*T*_g_) values for the obtained polymeric materials.

*T*_g_ [°C]								
PCM	PS	PGM	Copolymer 1	Copolymer 2	Copolymer 3	Copolymer 4	Copolymer 5	Copolymer 6
3.1	61.9	24.1	0.4	−7.7	−15.2	−29.0	−23.2	−50.5

**Table 7:** The thermogravimetric/derivative thermogravimetric (TG/DTG) data (helium).

Polymer Name	*T*_5%_ [°C]	*T*_max1_/*T*_max1a_ [°C]	Δ*m*_1_ [%]	*T*_max2_ [°C]	Δ*m*_2_ [%]	*m*_r_ [%]
PCM	251	396	99.0	577	1.0	0
PS	200	222	11.6	413	88.4	0
PGM	189	235	54.0	434	46.0	0
Copolymer 1	187	205	17.4	412	82.6	0
Copolymer 2	148	257	38.0	407	62.0	0
Copolymer 3	172	277	34.9	403	65.1	0
Copolymer 4	159	189/254	54.1	431	35.8	10.1
Copolymer 5	142	180/238	56.9	424	40.9	2.2
Copolymer 6	144	212/276	59.4	433	36.9	3.7

**Table 8:** The thermogravimetric/derivative thermogravimetric (TG/DTG data) (synthetic air).

Polymer Name	*T*_5%_ [°C]	*T*_max1_/*T*_max1a_ [°C]	Δ*m*_1_ [%]	*T*_max2_ [°C]	Δ*m*_2_ [%]	*T*_max3_ [°C]	Δ*m*_3_ [%]
PCM	241	-	-	292/392	93.7	505	-
PS	234	167/225	7.9	391	92.1	-	0
PGM	211	239	44.2	427	41.4	520	-
Copolymer 1	181	195	17.9	393	78.1	464	4.0
Copolymer 2	163	185/246	53.0	398	39.1	482	7.9
Copolymer 3	172	273	39.2	401	52.6	484	8.2
Copolymer 4	147	182/249	75.4	416	14.2	501	10.4
Copolymer 5	149	234	57.0	414	29.4	494	13.6
Copolymer 6	119	227	65.9	419	25.0	488	9.1

## Data Availability

The original contributions presented in this study are included in the article. Further inquiries can be directed to the corresponding author.
